# Factors Influencing the Use of Biomedical Health Care by Rural Bolivian Anemic Women: Structural Barriers, Reproductive Status, Gender Roles, and Concepts of Anemia

**DOI:** 10.1371/journal.pone.0170475

**Published:** 2017-01-26

**Authors:** Rebecca M. Bedwell, Hilde Spielvogel, Diva Bellido, Virginia J. Vitzthum

**Affiliations:** 1 Department of Anthropology, Indiana University, Bloomington, Indiana, United States of America; 2 Kinsey Institute, Indiana University, Bloomington, Indiana, United States of America; 3 Instituto Boliviano de Biología de Altura, La Paz, Bolivia; TNO, NETHERLANDS

## Abstract

The persistently high prevalence of anemia in rural highland Bolivia argues for targeted iron supplementation. We evaluated the cultural, structural and behavioral factors that may facilitate or impede an anemic woman's decision to secure this biomedical treatment from a rural Bolivian health center.

**Methods:**

Non-pregnant women from a rural town and its surrounding region were tested for anemia. During phase 1 (n = 181), anemic women received a written recommendation for low-cost purchase of iron pills at the nearest health center. They were subsequently interviewed on their actions and experiences.

**Results:**

Estimated anemia prevalence among these non-pregnant women was 50% higher than the national average. Despite holding conceptualizations of anemia generally aligned with biomedical concepts, only 40% of anemic women attempted to obtain iron supplements from the health center. Town residents were about twice as likely to attempt to purchase pills as outside-town residents. Town women who were concurrently breastfeeding and menstruating, considered anemia most serious for women, and considered family health the shared responsibility of spouses were most likely to decide to purchase iron pills. Age, education, or native language did not negatively influence this health care behavior.

**Conclusions:**

Securing iron supplements involves individual trade-offs in the allocation of time, cost and effort. Nonetheless, suitably tailored programs can potentially harness local perceptions in the service of reducing anemia. Because of their comparatively high motivation to obtain iron supplements, targeting concurrently breastfeeding and menstruating women could have a positive cascade effect such that these women continue attending to their iron needs once they stop breastfeeding and if they become pregnant again. Because a sense of shared responsibility for family health appears to encourage women to attend to their own health, programs for women could involve their spouses. Complementing centralized availability, biomedical and traditional healers could distribute iron supplements on rotating visits to outlying areas and/or at highly attended weekly markets.

## Introduction

Anemia, the low concentration of hemoglobin in the blood, affects as many as 2 billion people, including nearly one-third of all non-pregnant women, and accounts for about 9% of the total disability from all conditions worldwide [1]. Steady (and even increasing) rates of anemia are a formidable global public health challenge, especially in light of the disappointing results from many previous intervention efforts [2–4]. Anemia is most prevalent in less developed countries (LDCs), in which about half of all cases are caused by iron deficiency, arguably the most common nutritional deficiency in the world [1, 2, 4].

Some 100 million persons in Latin America and the Caribbean (LAC; comprising 32 countries) are anemic, with rural Bolivian women having among the highest anemia prevalence rates in this region [4]. A recent evaluation concluded that anemia is a significant health problem in most LAC countries for which data are available [5]. A comprehensive assessment by the Pan American Health Organization (PAHO) found that, despite national efforts including iron fortification of wheat flour, very little progress had been achieved in LAC over the previous two decades in reducing anemia rates among young children and non-pregnant women of reproductive age [4]. Although the PAHO report noted that there had been some reduction of anemia prevalence in pregnant women, anemia in this demographic segment continued to be a “severe public health problem” in 7 LAC countries (including Bolivia) and a “moderate threat" in 24 others [4]. At least 24% of women of reproductive age in LAC are anemic. In Bolivia the prevalence is even higher: 36.0% of urban and 42.3% of rural women are anemic [4].

Women of reproductive age and young children are the most vulnerable to developing anemia. Anemia is associated with poor pregnancy outcomes including maternal and offspring death, poor motor and mental performance in children, and low work productivity in adults. Lower current work productivity in anemic adults and lower future work productivity in anemic children have serious economic implications for LDCs [6].

Low bioavailable iron intake and absorption are major factors contributing to iron deficiency anemia, and iron fortification of food staples has often been considered the most effective strategy for addressing these factors [3,7,8]. Large-scale food fortification programs have been undertaken worldwide and throughout LAC to combat anemia. However, significant shortcomings of this approach include, among others, incompatibility between iron compounds and food matrices that limit the amount of fortification, and insufficient consumption of the fortified food [3,9]. For example, in 16 of 24 LAC countries, the average daily intake of iron-fortified wheat flour is inadequate for alleviating anemia [4]. Food fortification is clearly one valuable tool for alleviating nutrient deficiencies [3,9]. But an underappreciation of the inherent limitations of iron fortification has tended to encourage over-reliance on single-food fortification at the expense of the development and implementation of strategies to remedy the several determinants of persistent anemia [4].

Multiple integrated strategies are needed to achieve substantial reductions in anemia. PAHO [4] has recommended several ambitious yet achievable measures including improvements in iron supplementation programs and nutritional education programs using behavior change communication (BCC). However, interventions reliant on the participation and adherence of individuals can be challenging because, for a variety of reason, clients do not necessarily follow the instructions given by health personnel [2,10]. Tailoring such interventions to local cultures and living conditions (e.g., attending to the specific factors that may sway individuals' decisions regarding treatment) would likely improve these programs' efficacy and encourage individual adoption and sustained adherence.

Unfortunately, in stark contrast to the extensive study of various biological aspects of hemoglobin concentrations (e.g., [11–14]), there has been far less research on people’s conceptualizations of anemia or on how these beliefs might influence their health care behavior. Nonetheless, this work has revealed how local cultural constructs and practices can facilitate or impede the use of iron supplementation [2,10,15–21]. The few such studies in Bolivia have been conducted in urban centers and smaller cities [15–17]; none have investigated rural *altiplano* populations, in which the prevalence of anemia is particularly high.

In our study we sought to document and better understand the cultural, structural and behavioral factors that may be contributing to the persistently high prevalence of anemia in rural *altiplano* communities about 100 km south of La Paz, Bolivia. Livelihoods there have changed little since the agrarian reform during the 1950s. Most families in this region are Aymara speakers dependent on agropastoralism (hand-farming potatoes and perhaps barley or wheat, and herding sheep and/or dairy cows) [22–24]. The typical diet (inadequate bioavailable iron and other micronutrients, seasonally low energy intake, and very low dietary fat intake [25]) has not changed substantially over the past 20 years.

We assessed anemia status during two phases of data collection from non-pregnant women of reproductive age; in both study phases, anemia prevalence was above the national average. During the first phase of our study, we discussed the causes, symptoms, and negative health consequences of anemia with each study participant and, if anemic, encouraged her to obtain iron pills, available at very low cost at the local health center. Yet fewer than half of those women who were given a recommendation tried to obtain these supplements. This low rate of seeking biomedical treatment prompted our inquiry into the factors that may have influenced these women's behaviors.

Several novel findings emerged from our analyses, most notably that the reproductive status of non-pregnant women (i.e., whether a woman was breastfeeding *or* menstruating *or* menstruating+breastfeeding) was a strong predictor of attempting to purchase iron supplements. In women who were concurrently breastfeeding and menstruating, the probability of accessing biomedical treatment for anemia was more than double that of women who were either breastfeeding or menstruating but not both. In other words, the real and perceived health care needs of these non-pregnant women are not homogeneous. Programmatically, recognizing and harnessing this variability among non-pregnant women may help to reduce anemia at all stages of woman's life (a point we consider further in the Discussion). Amongst our study's participants, perceptions of the personal impact of anemia and of one's responsibility for family health also factored into the decision to obtain iron supplements. At the risk of stating the obvious, these culturally shaped values and ideas regarding health needs, risks, and treatments are inevitably shared by mothers with their children, especially their daughters. Thus, as witnessed by the iron-poor diets and persistently high prevalence of anemia, these perceptions and practices persist cross-generationally and continue to be salient in this rural agropastoral population.

Our analyses also suggested that trade-offs between the time and effort that must be devoted to economic activities, home and family versus that which would be needed to obtain and use iron pills influenced the decision to obtain iron pills. Those women living more distant from the health center were only about half as likely to attempt to purchase iron supplementation as were town residents. But even among town women, only 60% sought to purchase iron pills at the health center. Proximity is obviously not sufficient to ensure the use of health care services, a fact that highlights the importance of assessing which other cultural, structural, and behavioral factors in this region are influencing this complex decision. On this point, it is noteworthy that our analyses found that some factors that have impeded access to health care in other settings had little or no import in this rural *altiplano* population.

Although we met our study's objective to identify some of the locally salient barriers and facilitators affecting an anemic woman's decision to access iron supplementation, more in-depth research is still needed in this region. It remains to be seen if factors newly recognized in this population will prove, upon further study, to be influencing health behavior in other settings. Most importantly, steps must be taken to develop more effective programs to reduce the high prevalence of anemia among all rural Bolivian *altiplano* women. We note some possible options towards this goal in this paper's final section.

## Methods

Study participants were recruited from Patacamaya (a rural town located at the crossing of two highways about mid-way between La Paz and Oruro) and from surrounding small communities and homesteads scattered over approximately 400 km^2^. All study protocols, including consent procedures, were approved by the Consejo Scientifico, Instituto Boliviano de Biología de Altura, La Paz; the Institutional Review Board, University of California, Riverside; the Institutional Review Board, Indiana University. In accordance with cultural norms in the study population, verbal informed consent was obtained from all study participants. Giving, or declining to give, verbal consent was documented in confidential field records.

Most families in this region are indigenous Aymara agropastoralists (farmer-herders) primarily reliant on potatoes, barley, sheep and/or dairy cattle [22–24]. The only formal biomedical health care available throughout this region is in Patacamaya. A better-equipped secondary hospital has now replaced the smaller health center present during the first phase of this study. There are also traditional health care providers (*curanderos*) in the region; in this paper, health care refers only to the biomedical services provided in Patacamaya.

The single health facility in Patacamaya is a substantial distance both horizontally and vertically (two or more hours' walk) from most women’s homes in the surrounding rural area. For long distances, motorized transport has to be arranged and usually paid for. Few women have access to (or can drive) motorized vehicles or own (or can ride) a bicycle. Most of the time, most women in this region walk to get anywhere.

As recommended for populations at this study region’s average altitude (approximately 3800 meters), anemia in non-pregnant women was defined as having a hemoglobin concentration (Hb) less than 15.5 g/dL (see [13] for the determination of anemia thresholds in high altitude populations). Notably, many study participants often lived and worked at elevations greater than 3800m, but very few ever worked at lower elevations, therefore a threshold >15.5 g/dL is arguably more suitable for some individuals and would yield even higher estimates of anemia prevalence in this population. Hb of study participants was measured in 1996 (phase 1) and again in 2010 (phase 2); 75% of the women who participated in phase 2 had also participated in phase 1.

Individual Hb in phase 1 was measured using the HemoCue (Quest Diagnostics), a battery-operated portable point-of-care testing device that allowed us to measure Hb in each woman's home at a time arranged with her. It requires only a single blood drop from a finger prick. If found to be anemic at the time of measurement, a study participant was given a printed recommendation for a three-month supply of ferrous sulfate pills that could be obtained at the health center in Patacamaya at a cost of 1 *Boliviano* (about US$0.20 at that time). In a subsequent semi-structured private interview conducted in a participant’s native language by a trained female bilingual (Spanish/Aymara) Bolivian field assistant, participants were asked about their health-related beliefs and behaviors including the use of iron pills and experiences at the health center in Patacamaya.

Individual Hb in phase 2 was estimated by dividing hematocrit (packed cell volume) by 3 [26]. This method has acceptable accuracy in high altitude adult populations, as shown by studies in which both hematological indicators have been directly measured (e.g., [27]). We informed each participant of her anemia status and, if anemic, treatment options (e.g., dietary changes, seeing a physician) were discussed.

All statistical analyses were done with SPSS (v. 21) for Windows. Cross-tabulation, correlation, and logistic regression were used to evaluate associations between Hb and several factors hypothesized to influence Hb, and between an anemic woman’s decision to visit the health center for iron pills to treat her anemia and several factors thought to be influencing that decision; statistical significance was set at p≤0.05. Defined factors included locale (*town* = living in or close to Patacamaya, *rural* = communities several kilometers distant from town), reproductive status (*breastfeeding* or *menstruating* or *breastfeeding+menstruating* concurrently), first language (Aymara or Spanish), or education (i.e., completed years of schooling).

## Results

### Anemia prevalence and potential covariates of anemia

**Fig 1:**
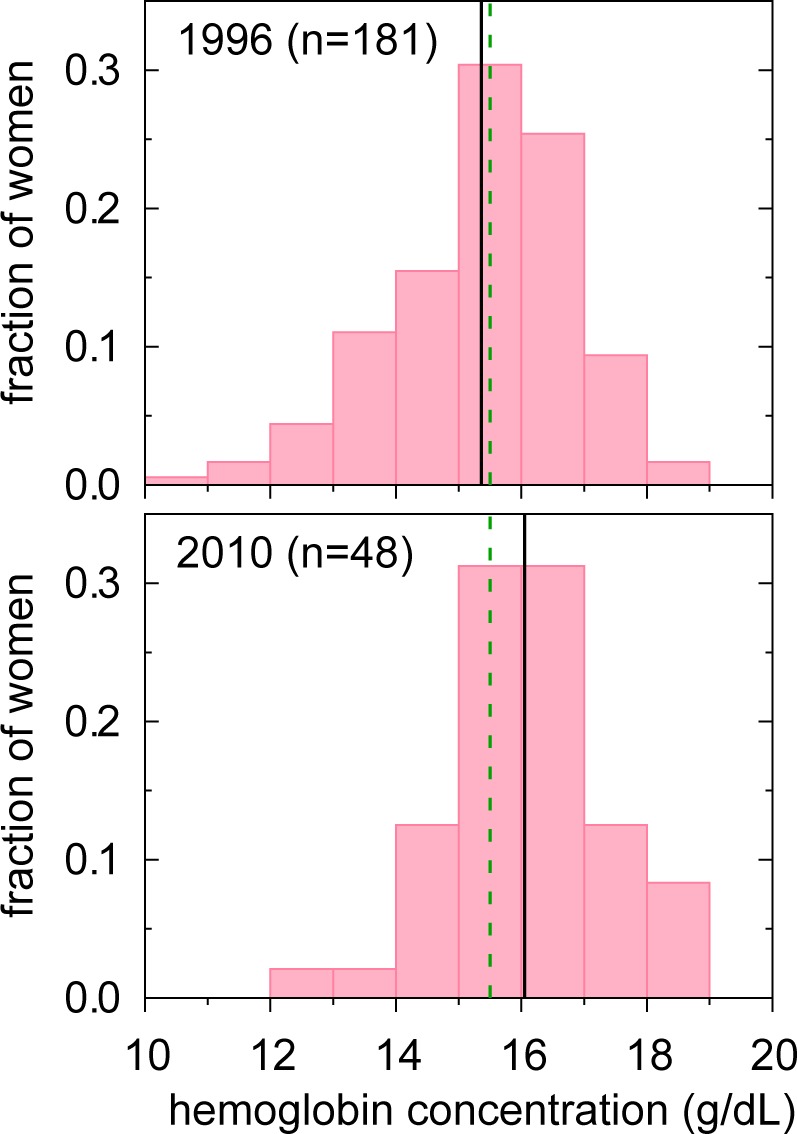
Hb Distribution in the Study Samples (Non-pregnant Women). Black solid vertical line = sample mean; green dashed vertical line = threshold for anemia (15.5 g/dL).

The samples for these analyses comprised 181 non-pregnant women in phase 1 with follow-up of 48 non-pregnant women (75% of whom had participated in phase 1) in phase 2. Fig 1 presents the distributions of Hb for non-pregnant participants in each phase of the study. In 2010 (phase 2), anemia prevalence (40% = 19/48 women) was *50% higher* than the national average for non-pregnant Bolivian women (26.4% in 2002 [4]). Furthermore, anemia prevalence in phase 2 was only 8% less than that observed in phase 1 (48% = 86/181 women), a difference that may be partly attributable to aging of these study participants in the intervening years and the fact that anemia is generally lower in older women [27].

Because of the larger sample size, we used the data from phase 1 (presented in Table 1) to evaluate whether Hb varied by locale, reproductive status, first language, or education. Hb and anemia prevalence did *not* vary significantly by any of these potential covariates nor by woman's age (23–36 years, mean = 28.6 ± 5.15 years; age also did not vary by any of these potential covariates). In sum, these attributes of the individual were not associated with her likelihood of being anemic, a finding that suggests anemia is not limited to some specific cohort of women but rather is a pervasive health problem throughout the region.

**Table 1:** Hemoglobin (Hb) Mean ± Standard Deviation (SD).

Analytical Samples	N	Hb (g/dL)	
		*M*	*SD*
Entire sample	181	15.4	1.48
Locale			
Rural	120	15.5	1.43
Town	61	15.1	1.56
Reproductive status			
Breastfeeding/not menstruating	68	15.3	1.41
Breastfeeding and menstruating	52	15.2	1.54
Menstruating	61	15.6	1.51
First language			
Aymara	147	15.3	1.51
Spanish	23	15.4	1.28
Education			
<6 years	114	15.4	1.55
≥6 years	54	15.2	1.35

### Use of biomedical treatment for anemia

Of the 181 non-pregnant women tested for anemia during phase 1, 100 were given a written recommendation to purchase iron pills: in 96 women, Hb<15.5 g/dL at the first or at a subsequent assessment; in 4 additional women Hb was 15.5–15.6 g/dL (these 4 recommendations were given because of notable weakness/fatigue and/or living at a higher than average elevation). Of these 100, 40 attempted to buy pills (but 2 were told at the health center that the pills were unavailable), 53 did not, and 7 declined to answer the question.

Of those who went to the health center in Patacamaya, the majority (61%) traveled on foot (Table 2). Most women (75%) were attended by a doctor, and most (82%) reported that interactions at the health center were in Spanish rather than Aymara. The median wait time was 20 minutes, but a few waited as long as an hour or more. When asked if they were treated well at the health center, 40% gave positive feedback, 26% responded negatively, and 26% responded “*No sé* [I do not know]” which may have meant either a neutral assessment of the treatment received or a way to avoid giving an explicitly negative assessment. After having waited, two women were told pills were not available at the center. Overall, fewer than half responded with a positive opinion of their experiences at the health center, and at least a quarter gave explicitly negative evaluations. In particular, rural women were significantly more likely than town women to report a negative experience.

**Table 2:** Reported Experiences at Health Center Organized by Locale of Participant.

Questions asked in interview	Participants by locale			
	All	Town^a^	Rural^b^	*p*^c^
Who bought the pills?	40	24	16	
Participant	80%	100%	50%	<0.0001
Husband	20%	-	50%	
Who attended participant at health center?	40	24	16	
Doctor	75%	92%	50%	0.014
Nurse	5%	-	13%	
Young woman	13%	8%	19%	
Don't know (husband bought pills)	8%	-	19%	
What language was used at health center?	40	24	16	
Spanish	82%	96%	62%	0.02
Aymara	10%	4%	19%	
Don't know (husband bought pills)	8%	-	19%	
How was the quality of care?	35	22	13	
Good/positive	40%	50%	23%	0.026
Poor/negative	26%	14%	46%	
"No sé"/ no opinion/neutral	26%	36%	8%	
Don't know (husband bought pills)	8%	-	23%	
Mode of transportation to health center?	38	23	15	
Foot	61%	96%	7%	<0.0001
Bicycle	13%	4%	27%	
Motorized vehicle	26%	-	66%	

^a^"Town": women living in or close to Patacamaya.

^b^"Rural": living in outlying communities.

^c^Significance for comparison of answers from rural vs. town samples.

Of those who obtained pills, only 39% reported having taken the pills for the recommended 3 months; the median duration was 2 months. In sum, of 100 women for whom biomedical treatment for anemia was recommended, only 14 reported completing the treatment regimen.

In light of the unacceptably high prevalence of anemia in populations throughout the world and especially at high altitude, and the seeming intractability of the problem in LAC despite various interventions [4], it is imperative that there is a more comprehensive and nuanced understanding of the factors that contribute to such a low rate of use of a simple, low client-cost, well tolerated and effective biomedical treatment for this common anemia. Below we examine those factors that may be of particular importance for the residents in and around Patacamaya.

### Does proximity affect use of health care?

As previously noted, most women in this region have limited transportation options, which suggests the simple hypothesis that proximity to health services is a major factor in a woman's decision to obtain iron pills (and perhaps other health care).

As expected, except for one woman who went by bike, all of the town participants who decided to get pills walked to the health center (Table 2). In contrast, half of the rural study participants who decided to get pills had their husbands go to the health center, and these men went by bicycle or motorized vehicle. Only one rural woman took the time to walk to the health center.

When those who did not attempt to obtain pills were asked why they did not go (open-ended response), the second most common reason given by rural women was not having enough time (24%), an answer consistent with having to walk several hours to get there and home again. In contrast, lack of time was not a common reason given by town women, who are within easy walking distance of the health center (Table 3).

**Table 3:** Reported Reasons for Not Obtaining Pills by Locale of Participant.

Reason for not obtaining pills	Participants by locale (%)		
	All (*n = 53*)	Town^a^ (*n = 16*)	Rural^b^ (*n = 37*)
Doesn't have time	19%	6%	24%
Doesn't have anemia	13%	6%	16%
Doesn't need the pills	2%	0%	3%
Forgot/lost recommendation	9%	13%	8%
Not given recommendation	44%	69%	32%
Other reasons	4%	6%	3%
Declined to answer	9%	0%	14%

^a^"Town": women living in or close to Patacamaya.

^b^"Rural": living in outlying communities.

**Fig 2:**
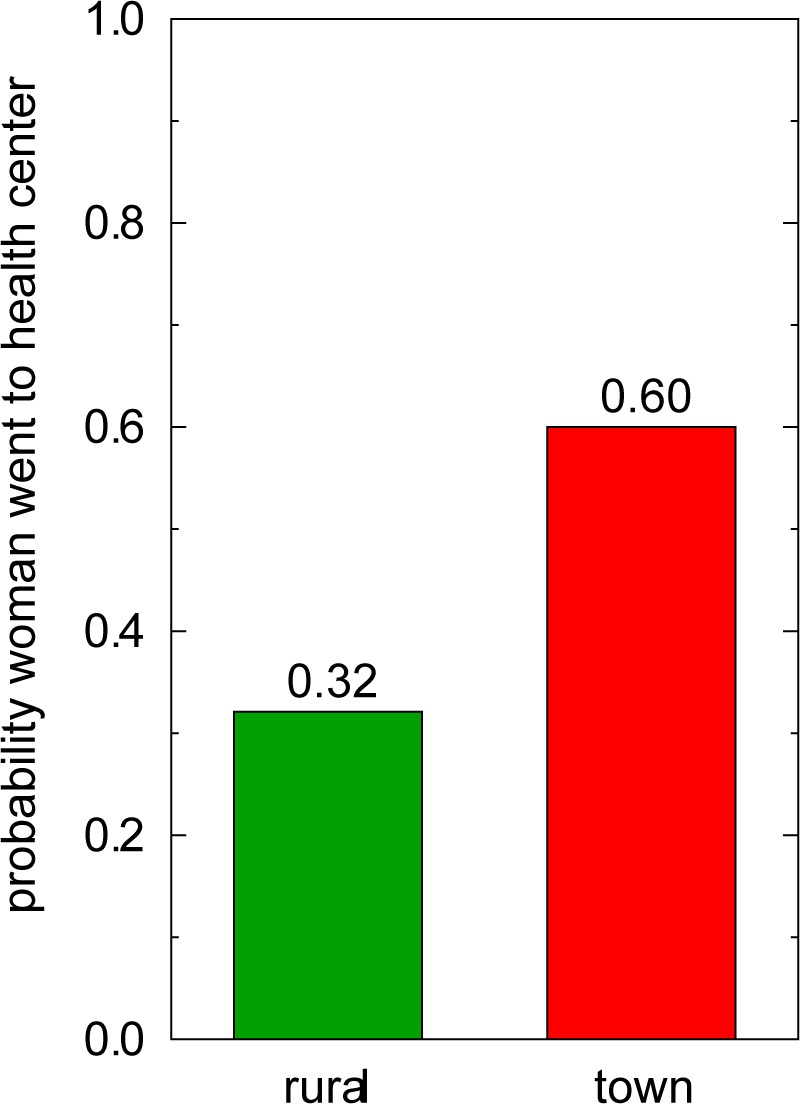
Probability of Attempting to Secure Iron Pills. Women living in or close to Patacamaya ("town") *vs*. those living in outlying communities ("rural").

We conducted logistic regression analyses to evaluate the hypothesis that proximity to services is important in the decision to obtain iron pills (Table 4, Model 1; Fig 2). The probability of seeking iron pills at the health center is 0.60 in town women, but drops to about half that (0.32) in rural women. In fact, although additional analyses (discussed below) confirmed several other factors to be significantly associated with town women's decisions to obtain pills, none of these appeared to have played a significant role in rural women's decisions. In other words, some aspect(s) of rural residence (arguably, distance to the town's health center, few transportation options, and the demands of agropastoralism, which limit the time available for such trips) appears to have over-ridden any other consideration (of those examined in this study) as to whether to obtain biomedical treatment for anemia at the health center.

**Table 4:** Factors Affecting Decision to Obtain Iron Pills (Logistic Regression Models).

Model	Goodness of fit^a^	Variable^b^	*B*	*SE*	*p*	*Odds ratio*
Univariate Models						
1	65%	Locale	1.156	0.437	0.008	3.176
	(59%, 69%)	Constant	-0.172	0.218	0.43	0.842
Univariate Models (Town residents)						
2	68%	Language	1.398	0.684	0.041	4.049
	(71%, 63%)	Constant	0.342	0.342	0.317	1.408
3	68%	Family health responsibility	1.398	0.684	0.041	4.048
	(71%, 63%)	Constant	0.342	0.342	0.317	1.408
4	69%	Anemia is most serious for…	1.552	0.702	0.027	4.722
	(74%, 63%)	Constant	0.265	0.351	0.449	1.304
5	68%	Reproductive status	2.708	1.111	0.015	15.000
	(50%, 94%)	Constant	1.131	0.555	0.042	3.098
Multivariate Models (Town residents)						
6	75%	Reproductive status	2.951	1.181	0.012	19.127
	(88%, 56%)	Family health responsibility	1.69	0.816	0.038	5.418
		Constant	1.107	0.574	0.054	3.024
7	74%	Reproductive status	2.776	1.18	0.019	16.049
	(87%, 56%)	Anemia is most serious for…	1.722	0.815	0.035	5.597
		Constant	1.015	0.578	0.079	2.759
8	73%	Reproductive status	2.68	1.141	0.019	14.589
	(56%, 83%)	First Language	1.361	0.77	0.077	3.901
		Constant	1.063	0.568	0.061	2.896
9	75%	Reproductive status	3.233	1.331	0.015	25.368
	(71%, 81%)	Family health responsibility	2.171	0.968	0.025	8.77
		First Language	1.882	0.935	0.044	6.566
		Constant	1.152	0.63	0.067	3.165
10	72%	Reproductive status	2.933	1.251	0.019	18.776
	(74%, 69%)	Family health responsibility	1.52	0.858	0.076	4.572
		Anemia is most serious for…	1.52	0.858	0.076	4.572
		Constant	0.977	0.6	0.103	2.657

^a^Correctly predicted by model: Total % of sample (% of those who decided to buy pills, % of those who did not).

^b^Locale (rural v. town), Language (Spanish v. Aymara), Family health responsibility (one or neither v. both), Anemia is most serious for (others v. women), Reproductive status (breastfeeding or menstruating v. breastfeeding+menstruating concurrently).

### Do language or education affect use of health care?

Aymara is the native language for most residents in the study region. Most women speak at least some Spanish, but men are more likely (because of education, military service, and work experiences) to have a good command of the official language. Most interactions at the health center were conducted in Spanish, which might reasonably be expected to have posed a significant barrier to accessing health care for those who have a limited command of this language.

**Fig 3:**
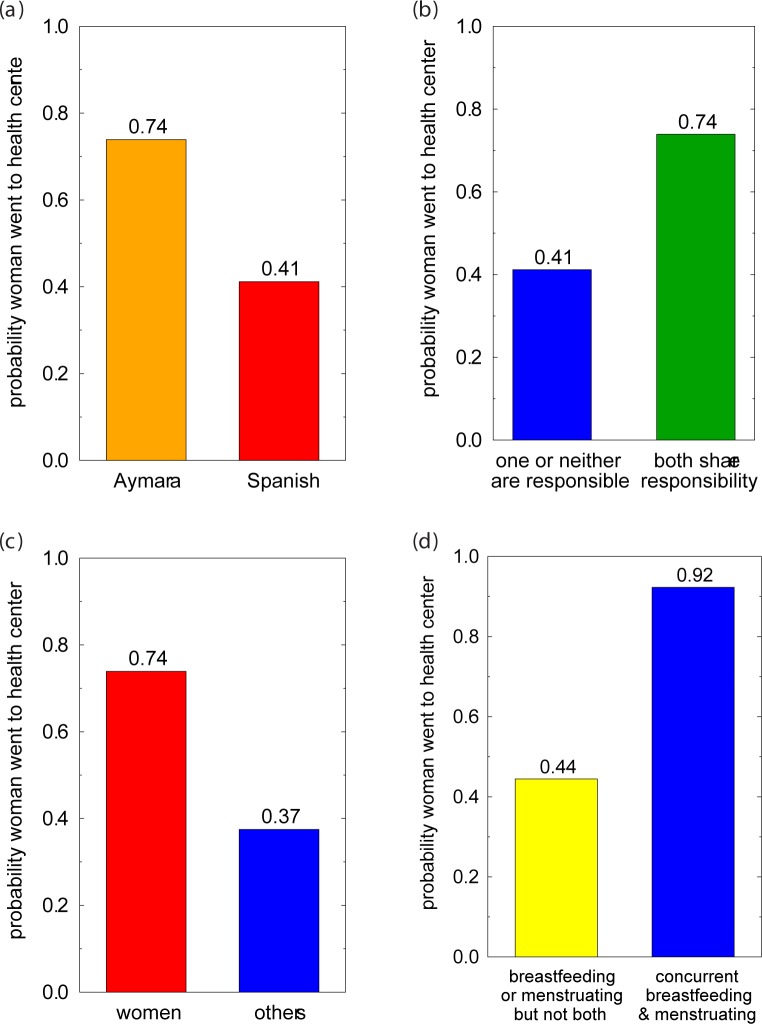
Factors Motivating or Impeding a Woman's Decision to Secure Iron Pills to Treat Anemia. Probability of town residents going to the health center for iron supplements by (a) first language, (b) opinion regarding which spouse (husband or wife) is responsible for family health, (c) opinion regarding for whom is anemia most serious, (d) reproductive status.

However, our data do not readily support this hypothesis. None of the women who did not try to obtain iron pills reported that the language spoken at the health center was an impediment. It may be that a woman was reluctant to report her lack of facility in Spanish. However, in the subsample of town women, the probability of trying to obtain iron pills was significantly *higher* among native Aymara speakers than native Spanish speakers (Table 4, Model 2; Fig 3A). We could not statistically analyze the effect of language for rural women because they were all native Aymara speakers. However, education level, which is positively associated with Spanish skills in this population, was not significantly associated with the decision to obtain pills in either the town or rural subsamples.

Collectively, our findings suggest that while the Spanish language may be a challenge for some Aymara speakers, it is not a major factor in their decision to obtain pills at the health center.

### Do gender roles affect use of health care?

Interviewees were asked, “Whose health is most important?”, and “Who is responsible for family health?” (categorical responses for both questions).

Most (83%) felt that the husband’s and wife’s health are equally important. This opinion did not vary by whether or not the participant had been told she had anemia nor by whether or not she had tried to obtain iron pills.

The majority of participants (67%) felt that spouses share responsibility for family health; 27% considered the wife to be primarily responsible. Those town women with a sense of shared responsibility were significantly more likely to obtain iron pills than those who believed otherwise (Table 4, Model 3; Fig 3B). Of 5 anemic women who felt that the husband was in charge of family health, only one bought iron pills.

These findings suggest that a sense of equality between spouses, at least regarding health, is most likely to foster the use of health care in town residents.

### Do conceptualizations of anemia affect use of health care?

In Aymara culture, blood is seen as the life force and is perceived to be a personal resource that is non-renewable during one’s lifetime [28]. Any blood loss is considered to be serious, and blood drawing is commonly rejected. Therefore, research team members explained and discussed anemia with each study participant before taking the first finger-prick blood sample for an Hb measurement. We described anemia as an illness of the blood that occurs when a person does not eat enough iron-containing foods. Enumerated symptoms included fatigue, weakness, headache, dizziness, and poor growth in children. Dietary recommendations included eating more iron-rich foods and drinking orange juice, but not coffee or tea, with iron pills (if recommended). Prior to our study, most persons in this region had not heard of anemia, therefore one goal of the project was to provide information about this significant health problem.

In the follow-up interview, we asked participants, “What is anemia?” (open-ended response), “How serious is it?” (scale response), and “For whom is it most serious?” (categorical response), in order to gauge the saliency of the biomedical information that had been presented and to learn about their conceptions of anemia as it relates to their own lives. Table 5 presents their responses to these questions.

**Table 5:** Reported Perceptions of Anemia Organized by Locale of Participant.

* *	Participants by locale [n(%)]		
Questions asked in interview	All	Town^a^	Rural^b^
What is anemia?^c^^,^^d^ (*n = 194*)			
Sickness	103 (53%)	29 (43%)	74 (56%)
Problems with eating/appetite	48 (24%)	27 (40%)	21 (16%)
Tired/weakness/lethargy	9 (5%)	8 (12%)	1 (0.8%)
Pain	19 (10%)	9 (13%)	10 (8%)
Other	10 (5%)	4 (6%)	6 (5%)
Doesn't know	44 (22%)	8 (13%)	36 (28%)
How serious is anemia?^e^ (*n = 166*)			
Very serious	105 (63%)	43 (74%)	62 (57%)
Somewhat serious	43 (26%)	12 (21%)	31 (29%)
Not serious	16 (10%)	2 (3%)	14 (13%)
Doesn't know	2 (1%)	1 (2%)	1 (0.9%)
For whom is anemia most serious?^e^ (*n = 168*)			
Women	99 (59%)	32 (53%)	67 (62%)
Babies	23 (14%)	12 (20%)	11 (10%)
Men	11 (7%)	2 (3%)	9 (8%)
Men and women	3 (2%)	-	3 (3%)
Women and babies	1 (0.6%)	-	1 (0.9%)
Everyone	29 (17%)	13 (22%)	16 (15%)
Doesn't know	2 (1%)	1 (2%)	1 (0.9%)

^a^"Town": women living in or close to Patacamaya.

^b^"Rural": living in outlying communities.

^c^Some women gave multiple answers, therefore total >100%.

^d^Rural v. town responses significantly different (p<0.001).

^e^Columns total 100%.

#### What is anemia?

Participants most commonly described anemia as a sickness (53%) and/or a problem with eating or appetite (24%). About one fifth said they didn't know what anemia is. A woman's own anemic status did not appear to influence her perception of anemia (anemic and non-anemic women did not differ in their descriptions [p = 0.60]). Moreover, a woman's perception of anemia did not appear to influence her decision to obtain biomedical treatment for anemia (women who did and did not try to obtain iron pills did not differ significantly in their descriptions of anemia [p = 0.08]).

Rural and town women differed significantly (p<0.001) in the set of responses to this question. Although large majorities in both regions described anemia as a sickness and/or a problem with eating or appetite, more than a quarter of rural women could not describe anemia, and rural women were more than twice as likely as town women to report that they did not know what anemia is (28% versus 13%). However, not being able to describe anemia was not associated with the decision to obtain iron pills in the sample as a whole or in either town or rural women.

#### How serious is anemia?

A majority of participants (63%) felt that anemia is "very serious." These women most commonly associated anemia with sickness (53%) and with problems with eating/appetite (44%). Appetite and diet are very important to Aymara concepts of health, so a lack of appetite is considered a serious issue [15]. Only 10% of the participants considered anemia to be "not serious." Somewhat counterintuitively to us, most (73%) of these women had nonetheless described anemia as an illness. Perhaps given the demands of life in the rural *altiplano*, an illness that does not result in the loss of blood and cannot be readily detected is not serious in the minds of some. There were no significant differences in the perceived seriousness of anemia between anemic and non-anemic women, or rural and town women, or those who did and did not attempt to obtain iron pills.

#### For whom is anemia most serious?

A majority of participants (59%) felt that anemia is most serious in women and about a fifth (17%) felt that anemia is equally serious for everyone. There was no significant difference between the opinions of anemic and non-anemic women. However, among town residents, anemic women who believed that anemia was most serious for women were almost twice as likely to visit the health center to obtain pills as those anemic women not holding this opinion (Table 4, Model 4; Fig 3C). Severity of anemia was not a factor in deciding to obtain pills: mean Hb did not differ significantly between those who did try to buy the pills (Hb = 14.1 ± 1.13 g/dL) and those who did not (Hb = 14.4 ± 1.05 g/dL).

In other words, those women with the most severe anemia were *not* more likely to try to obtain pills, but those women holding the opinion that anemia is most serious for women *were* more likely to try to obtain pills. These findings suggest that a belief can be more persuasive than a biological fact in the calculus underlying the decision to obtain biomedical health care.

### Does reproductive status affect use of health care?

Our finding that town residents who believed that anemia is particularly serious for women were very likely to try to obtain iron pills prompted us to hypothesize that women may recognize that their reproductive functioning makes them more vulnerable to anemia. Perhaps as a consequence of this insight, those women experiencing the greatest reproductive demands may be more likely to try to obtain pills than those experiencing less demands.

The results of our regression analyses support this hypothesis. Nearly all anemic town women who were concurrently breastfeeding and menstruating went to the health center to obtain iron pills; among other town women the probability of going to the health center was only 0.44 (Table 4, Model 5; Fig 3D).

### Undisclosed reasons for not securing iron supplements

Although we gave all anemic study participants a written recommendation for iron pills, 44% of those who had not purchased these pills stated their reason for not doing so was that they had not received this recommendation (Table 4). Town women were more likely to give this explanation than rural women, but overall there was no significant difference between these locales in the reasons given for not obtaining pills.

It is plausible that at least some anemic women did not recall having been advised to obtain iron pills. Perhaps some were seeking to avoid embarrassment at not having followed through on the advice, or some may have been masking reasons they preferred not to state to the interviewer. Our use of a questionnaire rather than in-depth interviews limits what can be concluded about why some anemic women reported that they had not received a written recommendation. On the other hand, probing the reasons behind this response could have violated socially acceptable conversational mores in Aymara culture. Additional questioning may have been seen as challenging a woman's veracity, or unwillingness of the research team member "to accept responsibility" for failing to provide a recommendation, or disrespectful of a person's right not to answer a question. Although we sense that there are undisclosed reasons for why women did not go to the health center to obtain iron supplements, it's not clear what these are likely to be.

### Multifactorial models of health care decisions

Obviously the various factors that promote or impede a woman's health care decisions operate simultaneously. Table 4 presents those multivariate models (6–8 are bivariate, 9–10 are trivariate) that had the highest goodness-of-fit to the data (i.e., the greatest explanatory power) in this study. As noted earlier, no variable other than locale was significantly associated with a rural woman's decision to try to obtain iron pills, therefore these multivariate models apply only to women residing in town.

**Fig 4:**
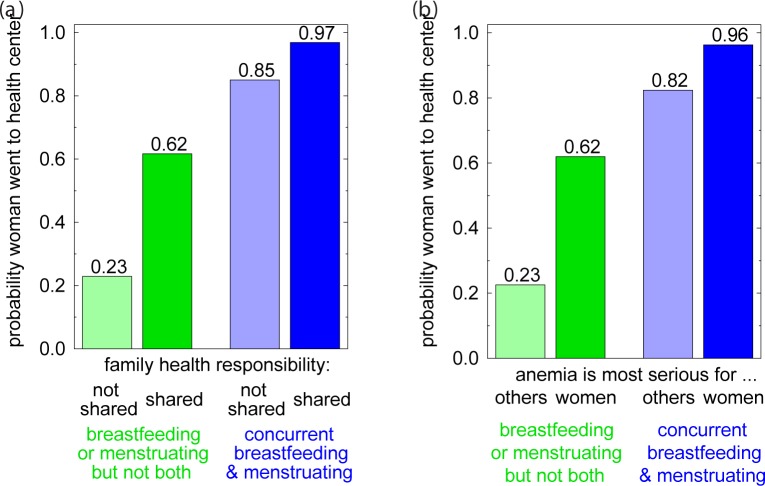
Multivariate Models of Factors Motivating or Impeding a Woman's Decision to Secure Iron Pills to Treat Anemia. Probability of town residents going to health center for iron supplements by (a) reproductive status AND opinion regarding which spouse (husband or wife) is responsible for family health [Model 6 in Table 4], (b) reproductive status AND opinion regarding for whom is anemia most serious [Model 7 in Table 4].

In every model, *reproductive status* is the single largest predictor of a town woman's decision. Compared to a model with only *reproductive status* (model 5), adding either *family health responsibility* (Model 6, Fig 4A) or *anemia is most serious for…* (Model 7, Fig 4B) increases the goodness of fit by 6–7%. In a trivariate model including all three of these variables (Model 10), both *family health responsibility* and *anemia is most serious for*… are not significant at 0.05 and the goodness-of-fit is slightly less than that of models 6 and 7. *First language* is not significant at 0.05 when paired with *reproductive status* (Model 8), but is significant in a trivariate model that includes *reproductive status* and *family health responsibility* (Model 9).

Model 9 is the "best model" by goodness of fit criteria (it predicts 75% of the sample overall, 71% of those who did try to obtain pills, and 81% of those who did not try). Model 9 predicts that those women who are concurrently breastfeeding and menstruating, and who hold the opinion that family health is the responsibility of both spouses, and whose first language is Aymara have the highest probability of visiting the health center to obtain iron pills. It seems unlikely that native Spanish speakers were deterred from visiting the health center; perhaps *Aymara speaker* in Model 9 is acting as a proxy for a suite of Aymara cultural values that encourage a woman to attend to her own health.

Collectively, the results of the logistic regression models presented in Table 5 suggest that in this rural *altiplano* population, structural barriers (proximity, time and cost of transport) are over-riding factors influencing the decision to obtain iron pills, and that for those near the health center, perceptions regarding who is most impacted by anemia, her role in family health decision-making, and her own reproductive status contribute significantly to a woman's decision to seek biomedical treatment for anemia and perhaps other health needs.

## Discussion

Anemia continues to be a major health burden for residents of the Bolivian *altiplano*, particularly in rural areas. In 2010, 40% of the participants in our study sample were anemic, which is 50% higher than the Bolivian national average for non-pregnant women. There has been little reduction of anemia in this demographic segment despite national efforts to fortify wheat flour. Health services in Patacamaya have improved over time, but residents in the surrounding communities still face much the same demands on their time and impediments to access as before, and the diets of most persons in this region still lack adequate bioavailable iron. The persistently high prevalence of anemia in this rural population argues for targeted iron supplementation and BCC interventions.

Our findings offer novel insights into Bolivian *altiplano* women's decisions to use biomedical health care to treat anemia. Knowledge of why women did or didn't choose to make use of an inexpensive biomedical treatment for anemia provides a valuable foundation for developing more effective programs to alleviate the problem in our study region and may also inform anemia intervention strategies elsewhere, especially in LDCs.

It is disconcerting that in this study only 40 of 100 anemic women attempted to obtain the recommended iron pills, despite the low cost to them for a 3-month treatment, and only 14 women completed the 3-month regimen. Logically, efforts to ameliorate anemia would be more effective if programs could address the reasons that women decide not to visit a health center rather than expecting women "to comply" with programs that may not suit their circumstances.

In the region served by the health center in Patacamaya, locale was the major determinant in obtaining biomedical treatment for anemia and is likely to still be a factor in seeking biomedical health care more generally. Anemic women living in the small communities and scattered homesteads around Patacamaya were only half as likely to attempt to purchase iron pills as those living in town. Proximity to the health center, time, and access to motorized transportation were likely major reasons behind this difference. The region has long lacked much, if any, public transport to the outlying areas, and even today the taxis and microvans operating in Patacamaya are not necessarily economical or accessible for most residents far from the town. Unfortunately, despite the widely appreciated importance of proximity for accessing health service, structural barriers remain all too common (and neglected) in the rural areas of industrialized countries as well as throughout LDCs worldwide.

Among those women in the outlying communities who acknowledged having received a written recommendation to obtain iron pills, about half explained that they had not tried to obtain the pills because of a lack of time. It is telling that at recruitment only 9% of the study participants reported having gone to the health center during the previous six months (compared with 66% of all U.S. adults who see a health care professional in a six-month period [29]). The demanding work of agropastoralism, the principal occupation of virtually all women and men in these rural families, begins every day of the year before dawn and ends after dark. Animals must be milked and walked, often long distances, to pasture. Fields must be plowed, planted and harvested, usually with hand tools. Women must process foods for storage or sale, prepare meals, wash clothes, and tend to the house and children. It is frankly astonishing that the typical rural *altiplano* agropastoralist has time for much else, and no surprise that these pressing subsistence tasks typically supersede non-urgent trips to the health center.

Other structural barriers may also have influenced a rural woman's decision to obtain iron pills. For example, among those who did attempt to purchase pills, rural women were three times more likely than town women to explicitly report a negative experience at the health center. These poor experiences more readily become common knowledge in smaller outlying communities, perhaps negatively influencing the decisions of neighbors. However, of the women who had decided not to obtain pills, none gave a poor opinion of the health center as a reason for not going there. This finding from the questionnaire is particularly intriguing because some participants had, at one time or another, casually voiced negative opinions of various aspects of local biomedical health care. Perhaps the formality of an interview inhibited the expression of such opinions or perhaps, whatever their opinion, simple proximity was the over-riding consideration. Other than locale, none of the factors we evaluated played a statistically significant role in the decision to purchase pills among women living outside the town.

Other studies and reviews (for example, [2,15,17]) have also found that proximity to the health clinic, travel time and expense, and inconvenient clinic hours all factored in women's decisions to obtain iron supplements. Poor experiences at medical facilities also contribute to client reluctance to use health services [2,17]. For example, Quechua and Aymara pregnant women in Cochabamba (a major city in the Bolivian *yungas*, a forested stretch along the eastern Andean slopes that is lower, warmer and more humid than the *altiplano*) were discouraged by perceived mistreatment of women, institutional norms that disregarded women’s sense of modesty and emic physiology, and uncommunicative medical personnel [15].

Within the study sample of women living in Patacamaya, those most likely to decide to obtain iron pills were concurrently breastfeeding and menstruating, saw anemia as most serious for women, and considered family health the shared responsibility of both spouses. A woman's age or education level, or being a native Aymara speaker, did not appear to impede a visit to the health center.

Reproductive status is the single strongest predictor in these town women of a decision to obtain iron pills. In women who were concurrently breastfeeding and menstruating, the probability of accessing biomedical treatment for anemia was more than double that of women who were either breastfeeding or menstruating but not both (see Fig 3D). Previous studies have not evaluated whether variation in the reproductive status of non-pregnant women influences health care decisions, perhaps because the greatest programmatic focus has been, understandably, on pregnant women. However, women who are currently menstruating are the most likely to become pregnant unless they are using contraception. Our data suggest that women perceive the dual physiological demands of concurrent lactation and menstrual cycling, that this perception prompts health care seeking, and that giving additional attention to this group of women may be a particularly productive intervention strategy for reducing anemia in women of reproductive age.

Another important, and novel, predictor of health care behavior in these women reflects gender roles and responsibilities within families. Women in town who believed that both spouses are responsible for family health were more likely to go to the health center. It is interesting that a sense of female autonomy (or burden) regarding responsibility for family health was not as likely as shared responsibility to foster a woman's use of health care for herself. Perhaps a sense of sharing the responsibility for family health made it more likely that a woman would believe her own health is as important as the health of other family members, or perhaps their husbands were more encouraging about women seeking health care. “In many households women do not have the support of concerned family members who encourage them to go to the doctor or to take care of themselves. In contrast, men are more likely to be strongly pressured by other family members, particularly wives and mothers, to seek treatment” [30]. In Woldemicael’s [31] study of autonomy among women in Eritrea and Ethiopia, women who were involved in making day-to-day household decisions were more likely to seek antenatal care than women whose husbands made the final decisions. The author argued that this association is caused by the degree of autonomy a woman has to take charge of her own health. However, there was no distinction made between shared responsibility and individual responsibility, as we did in our analysis. The idea that female autonomy or greater gender equity within a household increases the likelihood that a woman will seek health care is supported by our findings.

Galloway and colleagues' [17] evaluation and comparison of conceptualizations of anemia in eight LDCs (including Bolivia) found considerable cross-cultural similarities regarding recognition of a suite of symptoms that are typical of anemia (e.g., “headache, dizziness, paleness, ‘decayed blood,’ ‘thin blood,’, ‘low blood,’ weight loss, and loss of appetite"(p. 539)). However, the majority of participants in all but one country did not know the biomedical name for these symptoms or that they collectively constitute a specific illness. Rather, women attributed these symptoms to inadequate and poor quality food due to poverty. Participants in Bolivia, Guatemala, and Honduras felt that anemia is serious and that it can be fatal for pregnant women and infants [17].

Likewise, majorities of both rural and town women in our study reported conceptualizations of anemia consistent with a biomedical model, but rural women were significantly more likely to report that they did not know what anemia is. However, this lack of knowledge was not associated with a decreased probability of attempting to purchase iron pills nor with a shorter duration in pill use. There were no significant differences between anemic and non-anemic women’s conceptualizations of anemia.

There may be important differences among women in their perceptions of anemia that were not captured in our study. A short questionnaire is inadequate for developing a comprehensive understanding of these women’s knowledge and conceptualizations of anemia, and a few patterns in the data might have been better understood if we had had the opportunity to conduct in-depth follow-up interviews. For example, in Aymara culture, an "illness" is generally considered "very serious," and a very serious condition is considered an illness. Of all respondents, 63% said that anemia is very serious yet only 53% identified it as an illness. Of those participants who said anemia is very serious, only about half said it was an illness; 44% associated it with problems with eating or appetite. Perhaps these women perceived the gravity of anemia, but saw it as something other than an illness (e.g., a loss of appetite attributable to witchcraft, which obviously cannot be treated with pills but rather requires the skills and knowledge of a traditional *curandero*).

Currie and Wiesenberg [30] discussed four dimensions of beliefs (causality, controllability, susceptibility, and seriousness) that influence women's decisions to use health care. In order to actually seek health care, a woman must first believe that her health experience constitutes an illness, that it is serious enough to warrant a trip to the health center, that she can afford the time and unpaid effort needed to make the trip, that she has the autonomy to do so, and that the experience at the health center is one she wants to have [30]. Our data suggest that those anemic women who perceived anemia to be very serious (for whatever reasons) were *not* more likely to attempt to purchase pills than those who did not have this perception. Viewed from the perspective offered by Currie and Wiesenberg [30], perhaps the seriousness of anemia does not sufficiently offset the time and effort of traveling to the health center. However, anemic town women who believed anemia to be most serious *for women* were almost twice as likely to visit the health center to obtain pills as those anemic town women not holding this opinion.

Collectively, these analyses reveal a nuanced and complicated picture of the factors influencing the use of health care services, specifically biomedical treatment for anemia, by a Bolivian *altiplano* woman. Her decisions reflect trade-offs between the time and effort that must be devoted to economic activities, home and family versus that which would be needed to obtain and use iron pills; her perception of the impact of anemia on her own health, especially if she is concurrently menstruating and breastfeeding; and her role in managing her family's health.

This greater appreciation of the experiences and perspectives of these women can be used to develop more successful anemia interventions. At the same time several authors have cautioned that a better understanding among researchers of cultural and/or local explanations of illness does not make individual behavior predictable or make individuals more likely to adhere to advice from medical personnel [10] and that “all long-term medical regimens face compliance problems” [2]. Nevertheless, the prevalence of anemia in rural *altiplano* populations is unquestionably and unacceptably high. The costs of anemia to individuals, communities and nations create an obligation to identify and ameliorate those barriers to access that are within the provenience of the health care system.

Because targeted populations may encompass diverse cultures, even within a single country, iron supplement and educational programs are more likely to be accepted by and effective for anemic individuals if due consideration is given to locally salient concepts of illness and anemia, and to a woman's own perceptions of her circumstances and the trade-offs in time, effort and cost that she must balance to achieve well-being for herself and her family It is essential to recognize that Bolivian women and men are deeply concerned with the health and well-being of themselves and their families, and that they face often difficult trade-offs in the allocation of time, cost and effort. Their decisions regarding health treatments make sense in the contexts of their lives and circumstances.

Suitably designed education and supplement programs can potentially harness local perceptions in the service of reducing anemia. For example, because of their comparatively high motivation to obtain iron supplements, targeting concurrently breastfeeding and menstruating women could have a positive cascade effect such that these women continue attending to their iron needs once they stop breastfeeding and if they become pregnant again. Likewise, because a sense of shared responsibility for family health appears to encourage women to attend to their own health, intervention programs for women should involve their spouses.

Finally, we emphasize that, rather than focusing solely on individual behaviors and beliefs, if structural factors that impeded health care utilization have not changed, it is time that they did. For example, rather than centralized availability, biomedical providers and *curanderos* could distribute iron supplements on rotating visits to outlying areas and/or be present at the weekly open market attended by almost everyone in the region.

These and other proposals (see [4]) are not easy to accomplish, but the current conditions that impede reduction in anemia must be viewed as challenges to be overcome rather than as insurmountable obstacles. Creative approaches to the burden of anemia in the *altiplano*, in the rest of Bolivia and elsewhere are needed and possible.
